# Cutaneous Ulcer Caused by Apixaban Treatment Is Resolved after Replacement with Dabigatran

**DOI:** 10.3390/medicina58050691

**Published:** 2022-05-23

**Authors:** Alessandro Medoro, Daniela Passarella, Donatella Mignogna, Carola Porcile, Emanuele Foderà, Mariano Intrieri, Gennaro Raimo, Pancrazio La Floresta, Claudio Russo, Gennaro Martucci

**Affiliations:** 1 Department of Medicine and Health Sciences “V. Tiberio”, University of Molise, 86100 Campobasso, Italy; alessandro.medoro@unimol.it (A.M.); daniela.passarella@unimol.it (D.P.); donatella.mignogna@studenti.unimol.it (D.M.); carola.porcile@unimol.it (C.P.); e.fodera@studenti.unimol.it (E.F.); intrieri@unimol.it (M.I.); raimo@unimol.it (G.R.); pancraziolafloresta@unimol.it (P.L.F.); 2 Orthopedics and Traumatology Unit, “A. Cardarelli” Hospital, Azienda Sanitaria Regionale del Molise, 86100 Campobasso, Italy; 3 UOC Governance del Farmaco, Azienda Sanitaria Regionale del Molise, 86100 Campobasso, Italy; 4 Anesthesia and Intensive Care Unit, “A. Cardarelli” Hospital, Azienda Sanitaria Regionale del Molise, 86100 Campobasso, Italy; gennaromart@libero.it

**Keywords:** novel oral anticoagulants, apixaban, dabigatran, adverse drug reaction, leg ulcer

## Abstract

Nowadays, novel oral anticoagulants (NOACs) have shown improved safety profile and efficacy compared to vitamin K antagonists in the prevention of thromboembolic events occurring during different pathological conditions. However, there are concerns and safety issues, mostly related to adverse events following interactions with other drugs, in real-world practice. We report the case of an 83-year-old woman who developed a non-bleeding leg ulcer not caused by trauma or other evident pathological conditions after 10 days of treatment with apixaban 5 mg/q.d. She was switched from apixaban to dabigatran and the leg ulcer rapidly improved and completely cicatrized in 40 days. The resolution of the ulcer and the toleration of dabigatran therapy suggest an apixaban-specific reaction; however, the pathological mechanism of ulcer onset is currently unclear. Careful evaluation of hospital databases of Molise region (Southern Italy) hospitals identified two similar cases between 2019 and 2021. These cases underline the necessity of careful post-marketing surveillance, considering the rapidly increasing number of patients treated with NOACs and patient’s risk factors such as old age, high polypharmacy rate, co-morbidities, and peculiar genetic background related to NOACs pharmacokinetic features.

## 1. Introduction

For many decades, warfarin, a vitamin K-antagonist anticoagulant (VKA), represented the mainstay of stroke prophylaxis in patients with atrial fibrillation (AF). The market launch (2008–2016) of the Novel Oral Anticoagulants (NOACs), a class of direct-acting drugs that are selective for one specific coagulation factor, determined the therapeutic replacement of VKAs: apixaban, rivaroxaban, and edoxaban are direct inhibitors of factor Xa; instead, dabigatran is a direct factor IIa (thrombin) inhibitor. During these years, NOACs become rapidly the treatment of choice in the prevention of venous thromboembolism, stroke, and systemic embolism in patients with non-valvular AF, and in the treatment of deep vein thrombosis (DVT) and pulmonary embolism (PE) [1,2]. 

Fixed dosing, predictable pharmacokinetics, and easy compliance to the therapy have been claimed as key advantages, counteracting challenges in the management of VKAs; indeed, due to their complex pharmacodynamic and pharmacokinetic profile, VKAs are characterized by less manageability, routine monitoring of coagulation parameters, and large inter-individual dose–response variability with narrow patient-targeted therapeutic window [3,4]. 

Different trials and meta-analyses have compared the safety and efficacy of NOACs vs. VKAs in the prevention of thromboembolic disease in AF and in the DVT and PE treatment, describing the non-inferiority profile in terms of efficacy, improved safety, and lower risk of severe adverse drug reactions (ADRs), such as cerebral hemorrhages [5,6,7,8,9,10,11,12]. Although the promise of more safety, uncertainties remain surrounding NOACs use; limited data on their safety and pharmacokinetic profiles are available especially in critical populations, such as elder people in polytherapy, patients with co-morbidities and/or peculiar genetic backgrounds, due to the still poor number of studies conducted on their metabolism, enzymatic and drug–drug interactions [13].

Post-marketing studies and meta-analyses have highlighted the potential occurrence of unpredictable safety signals. Like VKAs, NOACs show bleeding as the most common ADR, including gastrointestinal bleeding (GIB): it was described as a higher risk of GIB in patients treated with a high dose of NOACs compared to those treated with warfarin [14,15,16]. Beyond bleeding complications, different systematic reviews and meta-analyses assessed the potential risk of myocardial infarction, liver and renal injury, and nervous system disorders [16,17]. Cardiovascular safety shows considerable heterogeneity among oral anticoagulants, dabigatran seems to have a higher risk compared to warfarin [18]. Liver injury risk, as the rare case of jaundice due to intrahepatic cholestasis, is a recent safety issue that was undetected in clinical phases and emerged only from post-marketing analysis, especially for rivaroxaban [19]. Not only patients with chronic kidney disease, but other patients as well, could have a higher risk of developing acute kidney injury if the therapeutic range of anticoagulation is exceeded [20]. A considerable number of nervous system disorders are related to the NOACs (mostly dabigatran and rivaroxaban), such as cerebral infarction, ischemic stroke, and cerebrovascular accidents [16]. Moreover, neurologic symptoms such as imbalance, non-vertiginous dizziness, headache, diplopia, and confusion/disorientation were evidenced after treatment with apixaban [21].

Concomitant use of drugs affecting hemostasis could increase the risk of NOACs bleeding. These include aspirin and other antiplatelet agents, other anticoagulants, heparin, thrombolytic agents, selective serotonin reuptake inhibitors (SSRIs), serotonin-norepinephrine reuptake inhibitors (SNRIs), and nonsteroidal anti-inflammatory drugs (NSAIDs). Potential interactions with other drugs acting on NOACs metabolizing enzymes may cause both an increased risk of NOACs-related bleeding or a reduced antithrombotic efficacy [22,23,24,25].

In light of the aspects considered so far, it becomes crucial to detect, assess, understand, and maybe prevent all NOACs ADRs [26], not just the severe ones, but even the very rare ones, such as body rash, epistaxis, ecchymosis, gingival bleeding and other [27,28] or any negative drug–drug interactions [29,30,31] detected in real-world experience, considering, as discussed above, the clinical complexity of patients treated.

## 2. Case Presentation

An 83-year-old female was admitted to the Orthopedics and Traumatology Unit of the “A. Cardarelli” Hospital in Campobasso (Molise region, Italy) for a left hip osteoarthritis. During the period of hospitalization, an electrocardiogram (ECG) showed short AF episodes and non-specific abnormalities in the ventricular repolarization, confirmed by Holter ECG that recorded a sinus rhythm with a short run of AF with a rapid ventricular response, numerous supraventricular ectopic beats (SVE) and ventricular ectopic beats (VEBs), and couplets predominantly. Moreover, there was no evidence of rhythm pauses or ST-segment abnormalities. Echocardiographic analysis revealed normal left ventricular myocardial thickness with a preserved systolic ejection function (67%), dilated left atrium (70 mL volume), aortic valve sclerosis with mild insufficiency, and moderate mitral valve insufficiency. Right ventricular and atrial functions were preserved.

Hypertension and paroxysmal AF were diagnosed, and, consequently, the patient started this polytherapy: 1.25 mg bisoprolol, 10 mg amlodipine, 30 mg zofenopril calcium, 25 mg furosemide, and gastric protection with 15 mg lansoprazole daily. The patient was listed for hip replacement surgery, and, according to the most recent guidelines for the prevention of thromboembolism in patients with non-valvular AF [32], started the treatment with 5 mg/q.d. apixaban.

Two months later, laboratory tests to evaluate anticoagulant therapy effect showed normal coagulation tests (prothrombin time, PT: 112%; INR: 0.92; antithrombin III: 106%; activated partial thromboplastin time, aPTT: 34 s), normal renal function (creatinine: 0.73 mg/dL; creatinine clearance: 66.37 mL/min), and moderate high uric acid levels (8.1 mg/dL). 

The patient was evaluated by an anesthesiologist to plan the total left hip replacement surgery: she was in a good health state but reported a continuous and throbbing pain in the left leg caused by a non-bleeding and ulcerate lesion on the malleolus that occurred spontaneously (without direct trauma or other pathological conditions known to be the cause of the leg ulcer) 10 days after starting therapy with apixaban 5 mg/die (the total apixaban exposure was about 60 days). 

**Figure 1:**
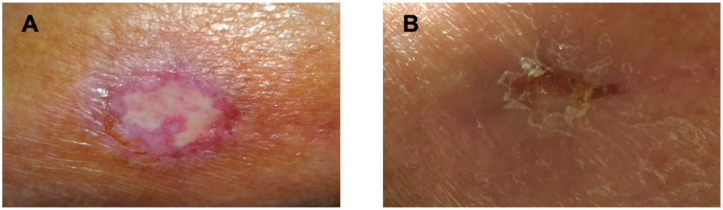
(**A**) Left malleolus discoidal ulcer (diameter of about 3 cm) characterized by central fibrotic and peripheral granulation tissues after 75 days of treatment with apixaban. (**B**) The lesion appears cicatrized 40 days after suspension of apixaban and replacement with dabigatran.

The lesion presented a discoid shape with a diameter of about 3 cm: whitish-colored fibrotic tissue was evident in the central portion of the ulcer; while on the peripheral side, there was a light red granulation tissue (Figure 1A). There was no evidence of infection or purulent or necrotic material, and the specialists excluded dermatologic diseases or insect stings, or animal bites, interpreting the lesion as a vascular alteration. The patient’s anticoagulant therapy was switched from apixaban to dabigatran, choosing the NOAC that pharmacologically acts with a different mechanism on the coagulation cascade.

The patient was evaluated again after 40 days of anticoagulant therapy with dabigatran, showing a normal chest X-ray and a good hemodynamic status, reporting normal atrioventricular and intraventricular conduction with isolated SVE and VEBs; the ulcerated lesion appeared almost completely cicatrized (Figure 1B). 

Some common mutations implicated in defective coagulation cascade were investigated: no known mutations were detected on factor V (R506Q, H1299R, or Y1702C) and factor II (G20210A); the patient has the 4G/4G genotype of plasminogen activator inhibitor-1 gene (*SERPINE1*) and carries the homozygous point mutations A1298C on the methylenetetrahydrofolate reductase gene (*MTHFR*). Although these mutations could be related to thrombosis events [33,34], no history of thromboembolism and/or recurrent pregnancy loss has characterized the patient’s clinical history. 

## 3. Discussion

We reported for the first time the case of a cutaneous ulcer (undetected not even in preclinical and clinical phases) [35] appearing in an elderly patient during treatment with apixaban. Ulcer onset occurred 10 days after apixaban therapy started. The patient was switched from apixaban to dabigatran: the ulcer resolution and the toleration of dabigatran therapy, without any new ADR, suggest an apixaban-specific reaction, considering that there are no other plausible causes as described above. Despite cutaneous ADRs being reported concerning rivaroxaban [28] and dabigatran [31], less is known about cutaneous reactions induced by apixaban. There is a single report, that, however, differs from our case: in 2016 was reported the case of a 78-year-old female that after 3 days of treatment with apixaban showed a psoriasiform eruption [36]. Interestingly, a careful evaluation of databases of patients in treatment with NOACs discharged from Molise region hospitals with a diagnosis of leg ulcer (ICD-9 codes: 707.10, 707.11, 707.12, 707.13, 707.14, 707.15, and 707.19) allowed us to identify two similar cases of which we do not have complete documentation: the case of an 85-year-old female with a huge right ankle ulcer (7 × 13 cm) appeared after a month of therapy with edoxaban in 2019 and the case of an 86-year-old female with multiple leg ulcers appeared after dabigatran replacement with rivaroxaban in 2021.

Following the Naranjo algorithm (setting a 6-point score for this report, Table 1) [37] and the WHO-UMC scale [38] to ascertain the causality assessment in pharmacovigilance, it is probable/likely that the leg ulcer onset in this case is caused by apixaban exposure.

Although the pathological mechanism of the cutaneous ulcer onset is currently unclear, we could hypothesize, considering the activity of apixaban, that the leg ulcer is caused by a coagulation defect linked to factor Xa and/or to the cascade of events downstream. In this case, this clinical report might indicate the possibility that some NOACs (apixaban in this case) could be able to unmask defects in the coagulation cascade linked to a peculiar genetic background, yet to be clarified. Furthermore, the patient’s 4G/4G homozygosity represents a minor variant of livedoid vasculitis, a rare vasculopathy that is typically characterized by painful lower limb lesions [39]; although the absence of patient clinical history of vasculopathy or thrombotic events and the quick recovery after pharmacological shift with a different drug, the occurrence of livedoid vasculitis cannot be completely ruled out. 

However, differences between apixaban and dabigatran concern not only their pharmacological targets but also their metabolization. As reported in the technical report, apixaban metabolism principally occurs via O-demethylation and 3-oxopiperidinyl hydroxylation primarily via CYP3A4/5 with minor contributions from CYP1A2, 2C8, 2C9, 2C19, and 2J2. Unchanged apixaban is the major form present in plasma, with no active metabolites in circulation. apixaban is also a substrate of P-gp transport proteins and breast cancer resistance protein (BCRP). Cytochrome P450 (especially CYP3A4/5) [40,41] and advanced age represent two of the contributing factors to the reduction in cytochrome P450 functionality [42]. However, we cannot exclude that an overexpression or an increase in CYP3A4/5 activity (on a genetic basis) significantly may have reduced apixaban levels in our patient, by inducing vascular thromboembolic microphenomena downstream. Dabigatran, instead, is primarily metabolized by esterases and cytochrome P450 plays no relevant role in its metabolism [43]. Considering the old age of the patient and her high polypharmacy rate, with drugs mainly metabolized by cytochrome P450 [44,45,46], these factors may have had a negative impact on the apixaban metabolism. 

This does not exclude that, in the context of polytherapy, other drugs may, in some way, have altered the absorption or metabolism of apixaban, considering the patient was taking bisoprolol, zofenopril, amlodipine, and furosemide for hypertension and lansoprazole for gastric protection. In the literature, there are some hints regarding bisoprolol and P-gp, and at least one evidence of interaction with dabigatran, although with contrasting evidence [47,48,49,50,51,52]. Therefore, considering that dabigatran itself is a substrate of P-gp, a further hypothesis is that a specific interaction regarding amlodipine, bisoprolol, or other drugs prescribed may have selectively altered the absorption (or excretion) of apixaban.

## 4. Conclusions

Our study highlights the onset of a rare clinical finding probably related to the administration of apixaban, an oral anticoagulant increasingly used in substitution of warfarin. The increasingly widespread use of NOACs is highlighting clinical problems, sometimes linked to pharmacokinetic or pharmacodynamic aspects or to interactions with other commonly used drugs, not found during the authorization studies. This focuses, on the one hand, on pharmacovigilance activities and on the phenomenon of underreporting, which is too widespread among health professionals, and on the other hand, the need for further studies to understand in more detail the metabolic aspects and unfavorable interactions with other drugs, likely underestimated to date. From this point of view, the lack of hematological references (for example, INR), although it represents a potential advantage for the uncomplicated patient, becomes a risk in fragile subjects, in polytherapy, or with complex genetic metabolic pictures still to be clarified.

## Figures and Tables

**Table 1:** Naranjo algorithm for the assessment of ADR. This questionnaire designed by Naranjo et al. [28] determines whether an ADR was caused by a drug or other factors. The ADR is assigned to a probability category from the total score as follows: “definite” if the overall score is 9 or greater, “probable” for a score of 5–8, “possible” for a score of 1–4, and “doubtful” if the score is 0. Bolded numbers apply to the patient case.

Assessment Questions	Yes	No	Don’t Know
1. Are there previous conclusive reports on the ADR?	+1	**0**	0
2. Did ADR appear after the suspected drug was given?	**+2**	−1	0
3. Did the ADR improve when the drug was discontinued, or a specific antagonist was given?	**+1**	0	0
4. Did the ADR appear when the drug was re-administered?	+2	-1	**0**
5. Are there alternative causes that could have caused the ADR?	−1	**+2**	0
6. Did the reaction reappear when a placebo was given?	−1	+1	**0**
7. Was the drug detected in any body fluid in toxic concentrations?	+1	0	**0**
8. Was the reaction more severe when the dose was increased, or less severe when the dose was decreased?	+1	0	**0**
9. Did the patient have a similar reaction to the same or similar drugs in any previous exposure?	+1	**0**	0
10. Was the ADR confirmed by any objective evidence?	**+1**	0	0
**Total Score**	**6 = Probable**		

## Data Availability

Data and material are available on reasonable request.
